# Nanomedicine-Based Strategies Assisting Photodynamic Therapy for Hypoxic Tumors: State-of-the-Art Approaches and Emerging Trends

**DOI:** 10.3390/biomedicines9020137

**Published:** 2021-02-01

**Authors:** Chun-Yan Shih, Pei-Ting Wang, Wu-Chou Su, Hsisheng Teng, Wei-Lun Huang

**Affiliations:** 1 Department of Chemical Engineering, National Cheng Kung University, Tainan 70101, Taiwan; n38081016@gs.ncku.edu.tw (C.-Y.S.); rosa110070523@gmail.com (P.-T.W.); hteng@mail.ncku.edu.tw (H.T.); 2 Center of Applied Nanomedicine, National Cheng Kung University, Tainan 70101, Taiwan; sunnysu@mail.ncku.edu.tw; 3 Department of Oncology, College of Medicine and Hospital, National Cheng Kung University, Tainan 70101, Taiwan; 4 Hierarchical Green-Energy Materials (Hi-GEM) Research Center, National Cheng Kung University, Tainan 70101, Taiwan; 5 Department of Medical Laboratory Science and Biotechnology, National Cheng Kung University, Tainan 70101, Taiwan

**Keywords:** nanomedicine, photodynamic therapy, hypoxia, nanoscale metal–organic frameworks, immunotherapy

## Abstract

Since the first clinical cancer treatment in 1978, photodynamic therapy (PDT) technologies have been largely improved and approved for clinical usage in various cancers. Due to the oxygen-dependent nature, the application of PDT is still limited by hypoxia in tumor tissues. Thus, the development of effective strategies for manipulating hypoxia and improving the effectiveness of PDT is one of the most important area in PDT field. Recently, emerging nanotechnology has benefitted progress in many areas, including PDT. In this review, after briefly introducing the mechanisms of PDT and hypoxia, as well as basic knowledge about nanomedicines, we will discuss the state of the art of nanomedicine-based approaches for assisting PDT for treating hypoxic tumors, mainly based on oxygen replenishing strategies and the oxygen dependency diminishing strategies. Among these strategies, we will emphasize emerging trends about the use of nanoscale metal–organic framework (nMOF) materials and the combination of PDT with immunotherapy. We further discuss future perspectives and challenges associated with these trends in both the aspects of mechanism and clinical translation.

## 1. Introduction

Cancer is one of the most concerning diseases all around the world, especially in developed countries [1]. Cancer’s wide variety made it a very hard-to-cure disease. Although recent advances in target therapies and immune therapies have proven to work quite well against cancer, it is still a long way to being effectively controlled [2]. In addition to conventional cancer therapy methods, such as surgery, chemotherapy, and radiotherapy, photodynamic therapy (PDT) has been developed and been approved for cancer treatment for decades [3].

### 1.1. Photodynamic Therapy

**Figure 1:**
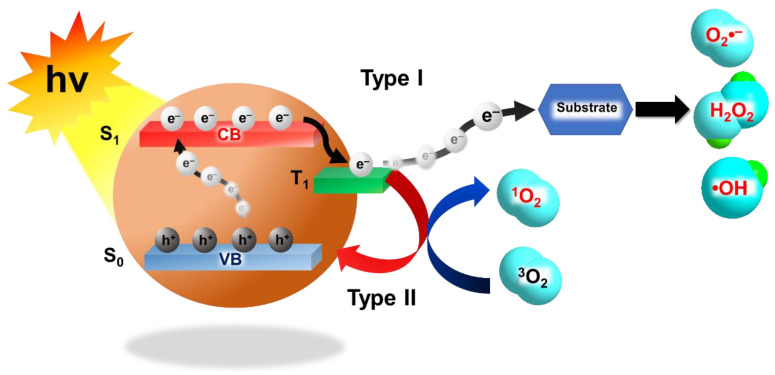
Schematic illustration of Type I photodynamic therapy (PDT) and Type II PDT. (hv: photon energy; CB: conduction band; VB: valence band).

Like surgery and radiotherapy (RT), PDT is a local treatment method, but it is less invasive and the side effects are relatively mild and not long-lasting [4,5]. PDT combines non-toxic photosensitizers (PSs), light illumination, and oxygen to produce reactive oxygen species (ROS), such as singlet oxygen (^1^O_2_), superoxide anions (O_2_^−^ •), hydroxyl radicals (OH •), and hydrogen peroxide (H_2_O_2_) to kill cancer cells. Typically, PDT can be divided into Type I PDT and Type II PDT (Figure 1). After absorbing photons during light illumination, PSs are activated from their ground state (S_0_) to a transient singlet excited state (S_1_) that can subsequently be converted to a long-lived triplet state (T_1_) through intersystem crossing. The type I mechanism describes the interactions of T_1_ electrons and the accompanying holes with surrounding substrates, such as water, biomolecules, and oxygen, to generate ROS like O_2_^−^ •, OH •, and H_2_O_2_. The type II mechanism is based on the transfer of the T_1_ electrons to the S_0_ state and accompanying energy release for ^1^O_2_ generation [6]. These ROS generated by PDT can oxidize different cellular components and induce different types of cell death, such as apoptosis, necrosis, and autophagy, according to the type, location, and amount of ROS generated [7]. Since the first clinical cancer treatment in 1978, PDT has also been approved for the clinical treatment of various cancers, including head and neck cancer, lung cancer, melanoma, colon cancer, bladder cancer, breast cancer, brain cancer, prostate cancer, liver cancer, and esophagus cancer [3,4,5,8].

### 1.2. Hypoxia

Hypoxia results from the inadequate supply of oxygen that compromises biologic functions. Tumor hypoxia is a hallmark of most advanced solid tumors, and is mainly caused by the aggressive proliferation of cancer cells and the abnormal vascular system in tumor area. Hypoxia has been shown to not only promote tumor metastasis but also have adverse impacts on different anticancer therapies, and play important roles in chemo-/radio-resistance [9,10]. Moreover, via the up-regulated, hypoxia-inducible factor-1α (HIF-1α), hypoxia has also been shown to facilitate immunosuppression and immune escape by activating lymphocytes, myeloid-derived suppressive cells, dendritic cells, and tumor-associated macrophages [11]. Although the oxygen concentrations in different types of human tumors are highly heterogeneous, generally the oxygen concentrations in the tumor tissues are much lower than those in the surrounding normal tissue. In many cases, many regions of tumors are even less than 5 mmHg partial pressure of oxygen (pO_2_), which corresponds to approximately 0.7% O_2_ in the gas phase or 7 μM in solution [12]. Thus, the efficiency of the anti-cancer strategies like RT and PDT that heavily rely on the existence of oxygen are largely hampered (the critical pO_2_ for RT and PDT are 25–30 and 15–35 mmHg, respectively) [10]. In the hypoxic area, a shortage of blood supply also lowers the efficiency of PDT by diminishing the penetration of PSs, which is worsen by the high expression of hypoxia-associated P-glycoprotein. Furthermore, the oxygen consumption by PDT itself would also reduce oxygen concentration, especially in cases that require continuous treatment. Thus, hypoxia can be the Achilles’ heel of PDT, and novel strategies are urgently needed [13].

### 1.3. Nanomedicines

In recent years, nanotechnological methods have been introduced in various different industries to provide a more efficient and effective alternative for solving a problem or providing a better product. Typically, nano-materials are defined as material with one or more external dimensions in the size range 1–100 nm [14]. Over the past decade, nanomaterials also developed rapidly and opened up new areas in biomedical applications for imaging, drug delivery, and various tumor therapeutic modalities, including photothermal therapy (PTT), radio-sensitization therapy, magnetic therapy, and also PDT [9,13,15]. For PDT application, in addition to leverage, the enhanced permeation and retention (EPR) effect for increasing the accumulation of PSs containing nanomedicines in tumor tissues, the large surface-to-volume ratios also effectively increase the number of PSs that can be delivered to the target cells. The nanomedicine delivery strategies also prevent the leakage of PSs and diminish the nonspecific accumulation of PSs in normal tissues that reduce overall photosensitivity [16]. Furthermore, a series of nanomedicine-based strategies have been designed for assisting photodynamic therapy for treating hypoxic tumors.

In this review, we will discuss the state of the art of the nanomedicine-based approaches for assisting PDT for treating hypoxic tumors, mainly based on oxygen replenishing strategies and the oxygen dependency diminishing strategies. Among these strategies, we will emphasize emerging trends about the use of nanoscale metal–organic framework (nMOF) materials and the combination of PDT with immunotherapy. We further discuss the future perspectives and challenges associated with these trends in both the aspects of mechanism and clinical translation.

## 2. Oxygen Replenishing Strategies

To restore PDT efficiency hampered by the shortage of oxygen in the hypoxic tumor microenvironment, replenishing the oxygen levels should be the most straightforward thinking. Oxygen levels can be replenished either by delivering oxygen via nano-carriers or generating oxygen in situ. In addition, modulating a tumor microenvironment, such as decreasing tumor oxygen consumption or improving blood flow, can also gradually restore local oxygen level.

### 2.1. Oxygen Carrying Strategies

Direct delivery of oxygen into tumor microenvironments is one of the most common approaches to overcome tumor hypoxia during PDT. It has been shown that the therapeutic efficiency of PDT can be enhanced by manipulating tumor oxygenation by hyperoxygenation or carbogen, not only in mouse models but also in clinical trials [17,18,19,20]. Recently, many types of oxygen nano-carriers have been developed.

#### 2.1.1. Oxygen Nano/Microbubbles

Free gas bubbles are not recommended to be injected directly into blood flow for hemolysis reasons. Instead, nano/microbubbles consisting of a stabilizing monolayered shell made by lipids or polymers and a gas core carrying oxygen have been shown to delivery oxygen for tumor oxygenation without side effects and enhancement of tumor therapy, including PDT [21,22,23]. Nano/microbubbles can also be loaded with drugs, either by encapsulation in the core or by coating the outer shell, according to the hydrophobic or hydrophilic properties [24]. In addition to direct administration, Huang et al. demonstrated that oxygen and PSs containing nano/microbubbles can be first internalized into tumortropic monocytes, and then be co-delivered via the tumortropic monocytes into the tumor microenvironment for improving PDT efficacy [25]. Furthermore, to avoid premature oxygen release and reduce side effects, the nano/microbubbles can be designed to control the oxygen release via external stimuli. Song et al. developed an acetylated, dextran-based, pH-responsive polymer to enable the release of oxygen preferentially in the tumor microenvironment [26]. Although significant progress has been made, the stability and storage issues of oxygen nano/microbubbles are still challenging.

#### 2.1.2. Artificial Red Blood Cells and Hemoglobin-Based Nanomaterials

Inside red blood cells (RBC), hemoglobin (Hb), which consists of four heme groups strongly binding to oxygen, is the natural carrier of oxygen in our body. However, free Hb is not a good candidate for delivering oxygen to hypoxic tumors, due to its low stability that would subsequently cause serious renal toxicity [27]. Thus, a series of Hb-based nanomaterials, often referred to as artificial RBCs, have been developed, and they have been shown to be highly potent for relieving tumor hypoxia and restoring PDT efficiency [28]. For example, Luo et al. developed stable, nano-sized artificial RBCs by loading complexes of Hb and indocyanine green (ICG), which serves as a PS, to incorporate an oxygen supply with PDT. The close distance between ICG and Hb by hydrophobic and electrostatic interactions in the artificial RBC enables self-enrichment of oxygen source for effective ROS production during PDT. In addition, the ROS subsequently induces the oxidation of ferrous Hb to become highly cytotoxic products and augment tumor destruction [29]. Although Hb has good biocompatibility, its limited oxygen transporting efficiency (only four oxygen binding sites for each Hb) restricts the application of artificial RBCs.

#### 2.1.3. Perfluorocarbon-Based Nanomaterials

Perfluorocarbons (PFCs) are organofluorines with the formula CxFy. Due to the extremely low polarizability of fluorine, PFCs exhibit great gas solubility, and their oxygen carrying capacity is 1.5 times higher than Hb [30]. In addition, PFCs have been approved for intravenous use by the United States Food and Drug Administration (FDA) as an artificial blood substitute [31]. Although PFCs have excellent oxygen carrying capacity, their super-hydrophobic features limit their development in tumor therapy. Fortunately, various nano-formulations, such as hollow nanoparticles (NPs), can solve this problem. For instance, hollow mesoporous silica NPs were used to load PFCs together with PSs or sonosensitizers, in order to generate ROS under near-infrared (NIR) light or ultrasound [32,33].

#### 2.1.4. Nanoscale Metal-Organic Frameworks

**Figure 2:**
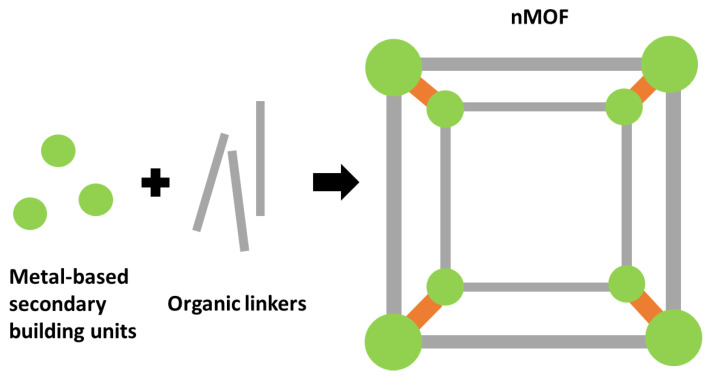
Schematic illustration of the construction of a nanoscale metal–organic framework (nMOF) from metal-based secondary building units and organic linkers.

As a kind of porous nanomaterial, with uniform pore size and large surface area (Figure 2), nanoscale metal–organic frameworks (nMOFs) have great capacity for gas storage, especially for oxygen [34,35,36]. In addition, based on the flexible cargo design, the multifunctional characteristics of nMOF make it a good choice of vehicle for oxygen-enhanced PDT, loading PS with oxygen [37,38]. The tunable feature enables nMOFs to be smart carriers that can preferentially release oxygen cargos in the tumor microenvironments. For examples, Lin et al. developed a novel multifunctional nanocarrier UC@mSiO_2_-RB@ZIF-O_2_-DOX-PEGFA, which combines oxygen-enhanced PDT with pH-responsive chemotherapy. The outermost shell, constructed with the zeolitic imidazolate framework 90, can be decomposed under acidic conditions, allowing preferential release of oxygen at tumor microenvironments with low pH, and therefore improving the PDT efficiency conducted by the co-delivered Rose Bengal PSs [38]. In addition to directly carrying oxygen, nMOFs are also capable to transport the PFC cargos for oxygen delivery [39].

### 2.2. Oxygen-Generating Strategies

Besides replenishing the oxygen levels in the hypoxic tumor microenvironment by direct delivery of oxygen, the oxygen level in the hypoxic tumor microenvironments can also be increased by in situ oxygen generation. This approach can avoid the side effects of oxygen toxicity caused by potential leakage via the oxygen-carrying strategies in the normal part of the body.

#### 2.2.1. Hydrogen Peroxide Decomposition

Besides the induction of hypoxia, the aggressive proliferation of cancer cells also results in the accumulation of ROS in the cancer cells. It has been shown that the H_2_O_2_ concentration is largely elevated in tumor tissues compared to the normal parts, which has also been linked to some key alterations in cancer, including cell proliferation, angiogenesis, and HIF-1 activation [13,36,40]. In normal tissue, catalase plays an important role to protect cells from oxidative damage from H_2_O_2_ by catalyzing the decomposition of H_2_O_2_ to oxygen and water [13]. Though free catalases have great efficiency in catalyzing the decomposition of H_2_O_2_ to generate oxygen, the instability of free catalases in vivo in the presence of proteases and poor half-lives have made the nanomedicine-based formulations, such as nMOF loading, essential for their application [41,42]. Leveraging the abnormal H_2_O_2_ concentrations in tumor microenvironments, many advanced nanomedicine-based PDT strategies have been designed to relieve the hypoxic condition and elevate the therapeutic efficiency of PDT by introducing a catalase to transform endogenous H_2_O_2_ to oxygen [43,44]. For example, Chen et al. constructed an αvβ3 integrin-targeting, H_2_O_2_-responsive, and O_2_-evolving NP to specifically eliminate αvβ3 integrin-rich tumor cells with elevated H_2_O_2_ levels. After specific uptake via the RGDfK–αvβ3 integrin interaction by cancer cells, the intracellular H_2_O_2_ penetrated the NPs, and was then catalyzed by catalase to generate oxygen. The continuously generated oxygen greatly improves the PDT efficacy in hypoxic tumors without damaging normal cells [44].

**Figure 3:**
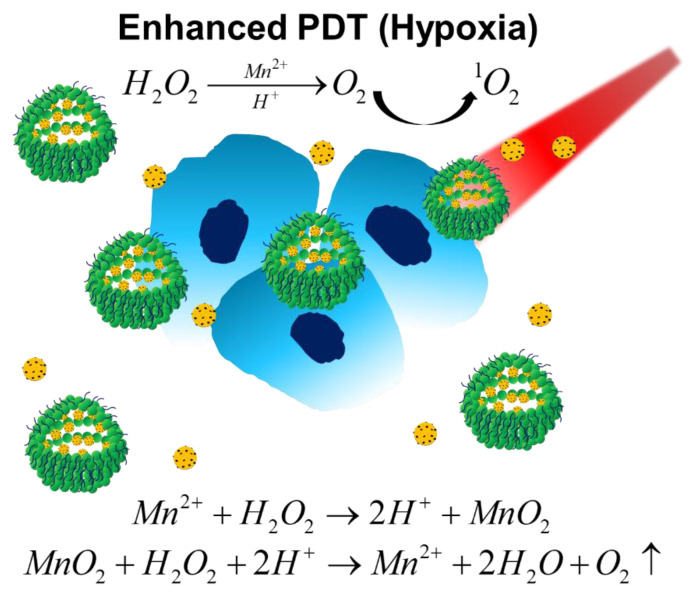
Schematic illustration of Mn–CD assembly catalyzing the decomposition of H_2_O_2_ to oxygen under acidic/hypoxic microenvironments, which exerts oxygen-enhanced PDT. Reproduced with permission [45]. Copyright 2018, Wiley-VCH.

Besides catalase, MnO_2_-, gold-, and platinum-based nanomaterials have been shown to exhibit similar catalytic function, and have been used in oxygen-enhanced PDT systems to catalyze the decomposition of H_2_O_2_ to replenish oxygen levels in hypoxic tumors and restore the PDT efficiency [13,45,46,47,48]. For example, Lin et al. recently developed a magnetofluorescent carbon dot assembly as an oxygenerator to relieve tumor hypoxia, which can exert fluorescent/ Magnetic Resonance Imaging (MRI) imaging and enhanced PDT simultaneously [45]. As shown in Figure 3, the nano-assembly was derived from manganese (II) phthalocyanine (Mn-Pc), followed by cooperative self-assembly with DSPE–PEG polymers to improve the solubility and biocompatibility. In addition to efficient ^1^O_2_ generation and tumor killing under normaxia, the Mn–carbon dots (CD) assembly can highly catalyze H_2_O_2_ to generate oxygen, and also successfully relieve tumor hypoxia for improving PDT efficiency. A two-fold increase in ^1^O_2_ production under irradiation was showed compared with that of counterpart NPs without MnO_2_. In addition, the oxygen levels increased 3.5-fold after intravenous injection of this nano-assembly.

#### 2.2.2. Water Splitting

Although catalase and the nanozymes are capable of catalyzing H_2_O_2_ to generate oxygen effectively, the endogenous H_2_O_2_ levels in the tumor microenvironments limit hydrogen peroxide decomposition strategies, especially in tumors with relative lower endogenous H_2_O_2_ levels [49,50,51]. Thus, some researchers tried to generate oxygen by decomposing/splitting water instead of H_2_O_2_ for replenishing oxygen levels in tumor microenvironments, which has already drawn a lot of attention in energy and environmental areas [52,53,54]. Some natural materials like thylakoid can be used to efficiently catalyze oxygen generation via water splitting. It has been reported thylakoid membrane-coated NPs re-oxygenate the hypoxic microenvironment and inhibit anaerobic respiration [55].

However, the extraction and preservation of natural materials is complicated, and the efficiency of oxygen generation is largely influenced by the physiologic environment. It is recognized that the well-known, metal-free, water-splitting material carbon nitride (C_3_N_4_) has the potential for biomedical applications. However, the absorption wavelength of pure C_3_N_4_ is in the ultraviolet and visible range, which limits its penetration depth with potential side effects, such as skin damage. Zheng et al. decorated a C_3_N_4_ nanocomposite with carbon dots that enhanced red light absorption and showed that the decorated nanocomposite efficiently produced oxygen from water [56]. Wang et al. also introduced the tungsten nitride (WN)-based nanomaterial that was originally designed for green energy application via water splitting to provide oxygen support to oxygen-enhanced PDT against hypoxic tumors [57,58]. Unlike semiconductor photocatalysts, WN with metallic properties could photocatalyze overall water splitting at longer wavelengths, up to 765 nm, which is suitable for in vivo oxygen production via water splitting due to the better light penetration. Although water-splitting materials used for generating clean energy in the energy and environment fields might also have the potential to be used for biomedical applications, the biocompatibility of these materials should be carefully deliberated.

#### 2.2.3. Self-Decomposition Compounds

Self-decomposition compounds, such as calcium peroxide (CaO_2_), gold oxide (Au_2_O_3_), and some platinum (IV)–azide complexes, which are mainly composed of metal, provide another safe and precisely controlled way to generate oxygen in situ in the tumor microenvironment [59,60,61]. CaO_2_ has been used for oxygen generating to relieve hypoxia induced by tissue necrosis within tissue-engineered implants in the early engraftment period [62,63]. Inspired by this concept, CaO_2_ has recently been introduced to synergistically work with PSs for oxygen-enhanced PDT efficiency. Liu et al. reported that the release of CaO_2_ from the damaged liposome-based NPs, caused by lipid peroxidation after laser irradiation, increased the contact of CaO_2_ to water and induced oxygen generation. The elevated oxygen improved the ^1^O_2_ generated by methylene blue (MB) under irradiation [59].

### 2.3. Tumor Microenvironment Modulating Strategies

Besides delivering oxygen to the tumor microenvironment or generating oxygen in situ, modulating a tumor microenvironment, such as decreasing tumor oxygen consumption or improving blood flow, can also gradually restore local oxygen levels.

#### 2.3.1. Improving Blood Flow

Since one major cause of tumor hypoxia is insufficient blood flow due to an abnormal vascular system in the tumor area, improving blood flow should be an effective approach to replenish oxygen levels in hypoxic tumors. It has been shown that a low concentration (1 μM−1 mM) of NO can relieve tumor hypoxia by modulating blood vessel relaxation to increase blood flow, decreasing tumor oxygen consumption rate, and accelerating the metabolism of intracellular glutathione (GSH) at the same time [64,65,66]. The great effects of NO on hypoxia relief have pushed the development of a series of strategies to achieve targeted delivery and the controllable release of NO [64,66,67]. For example, Wan et al. developed a tumor-specific, ROS-responsive NO generator nanoplatform by incorporating the NO donor l-Arginine (l-Arg) into a porous coordination network (PCN), which is a porphyrinic nMOF. Local ROS generation by a PCN under NIR led to the conversion of L-Arg into NO, which mediated the sensitized photodynamic therapy of the hypoxic tumor [68].

It has been also shown that tumor blood flow could be increased by mild heating [69,70]. Thus, pretreatment with mild heating by PTT is a favorable approach to relieve tumor hypoxia and regain the efficiency of PDT. To achieve the PTT heat-activated PDT, Feng et al. co-encapsulated a hexylamine conjugated chlorin e6 (hCe6) PS together with a lipophilic, NIR, DiR dye for PTT into PEG-shelled liposomes. A 785 nm laser was used to excite DiR-mediated PTT, followed by a 660 nm light-emitting diode that was used to trigger the hCe6 mediated PDT. The in vivo data showed that this protocol significantly improved intratumoral blood flow, which relieved tumor hypoxia and achieved the PTT heating-activated PDT [71]. In addition to using NO or mild heating to improve intratumoral blood flow, chemotherapeutic agents like taxane, gemcitabine, cyclophosphamide, and cisplatin also have been employed to modulate the abnormal tumor microvasculature for a more efficient blood and oxygen supply into tumors [72,73,74,75].

#### 2.3.2. Tumor Oxygen Consumption Decreasing

Besides directly increasing oxygen supply, diminishing oxygen consumption can also replenish oxygen levels in a tumor microenvironment. Respiration is the main mechanism that consumes oxygen in living cells [76]. Although aerobic glycolysis is the major metabolic pathway of tumor cells, respiration still plays an important role in tumor growth [77]. Thus, strategies for inhibiting respiration to decrease tumor oxygen consumption also have to be designed to solve the hypoxia issues in tumors. For example, metformin, a clinically approved type II diabetes drug, has been shown to alleviate tumor hypoxia by inhibiting respiration [78,79]. Song et al. co-encapsulated hydrophobic hCe6 and hydrophilic metformin into the outer membrane and inner cavity of the liposome. They showed that the sustained release of metformin greatly improved tumor oxygenation as well as the therapeutic efficiency of PDT in different tumor models [78]. Similarly, inhibiting respiration by NO or inhibiting mitochondrial oxidative phosphorylation with the oxygen regulator atovaquone have also been shown to overcome the hypoxia barrier and enhance PDT [80,81].

## 3. Oxygen Dependency Diminishing Strategies

In addition to replenishing the oxygen level in tumor microenvironments, alternatively, the therapeutic efficiency of PDT can also be achieved by strategies to diminish oxygen dependency. Thus, new paradigms that diminish oxygen dependency, such as type I PDT and fractional PDT, have recently become very popular. In addition, to enhance overall therapeutic efficacy, several other types of oxygen-independent therapies with potential synergistic effects have been used in combination with PDT, especially to overcome the hypoxic challenges in the tumor microenvironments.

### 3.1. Type I Photodynamic Therapies

As mentioned in Figure 1, in contrast to the type II PDT used in most existing PDT systems, which relies on the direct energy transfer from excited PSs to oxygen to generate ^1^O_2_, type I PDT can transfer energy from photosensitizers to other substrates like water and produce respective radical ions or radicals. Although the details of the operation of type I mechanisms are still under debate, many studies have reported that type I PDT has great efficiency, even under hypoxic conditions [82,83]. Thus, type I PDT might be a lead for designing new strategies to overcome the limits of hypoxia [84,85,86,87].

Titanium dioxide (TiO_2_) is widely used in nanomaterials, due to its high photocatalytic activity and biocompatibility. After absorbing UV light, TiO_2_ nanomaterials can oxidize the water to form OH by photogenerated holes, and exhibit good antitumor effects in animal models [88,89]. However, the penetration issues of UV limits its application. To solve the problem, many studies have tried to move the absorption of TiO_2_ nanomaterials to longer wavelengths with various decorations, such as carbon nanodots and upconversion nanoparticles, and have successfully demonstrated that near-infrared light triggers PDT [90,91]. Similarly, Lv et al. demonstrated excellent type I PDT with a high OH-based photocytotoxic effect under hypoxia using the cyclometalated Ru complex [84]. In this study, a coumarin group was covalently incorporated into this complex to increase its light-harvesting ability, and also to enable it to serve as a good electron donor, which is critical for type I PDT.

### 3.2. Fractional Photodynamic Therapies

Due to the oxygen consumption during PDT process, PDT itself is a course of hypoxia. Thus, the PDT efficiency will be gradually decreased after continuous irradiation. To overcome this problem, researchers have tried to alter the irradiation protocol that introduces breaks between irradiation periods for the replenishment of intracellular oxygen. Many studies suggest that fractional (intermittent) delivery of light might be a better approach to PDT [88,90,91,92,93,94]. For example, Xiao et al. showed that the fractional irradiation protocol of 100 s on/600 s off cycles have better therapeutic efficiency than the continuous irradiation protocol and the 1 s on/6 s off cycles with the same light energy input in rat prostate cancer model [90].

**Figure 4:**
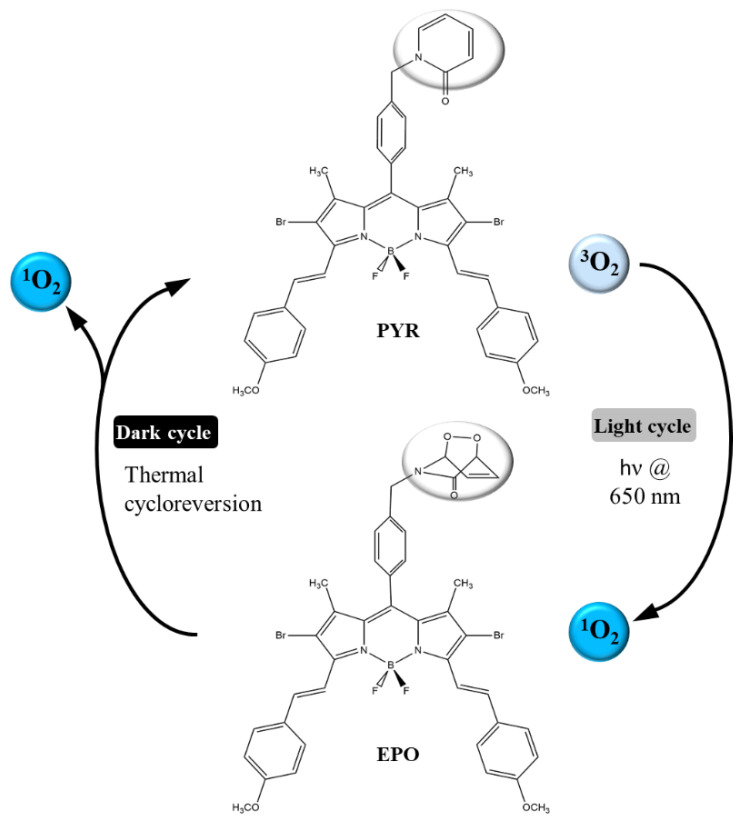
Schematic illustration of the mechanism of the novel fractional PDT photosensitizer (PS) based on the 2-pydidone-conjugated boron–dipyrromethene (BODIPY). Reproduced with permission [92]. Copyright 2016, Wiley-VCH.

In addition to the approach of editing irradiation protocols, efforts toward PS improvement have also been ongoing. To further enhance fractional PDT, Turan et al. developed a boron–dipyrromethene (BODIPY)-based PS [92]. In this system, BODIPY is incorporated with 2-pyrdidone to a bifunctional compound (PYR), in which 2-pyridone and its endoperoxide (EPO) derivative undergo a reversible recovery reaction that releases ^1^O_2_ efficiently (Figure 4). Upon light irradiation (light cycle), Pyr produces ^1^O_2_, so that some of it is absorbed by reaction with 2-pyridone to form endoperoxide. When irradiation is stopped (dark cycle), EPO releases ^1^O_2_ and regenerates PYR. Therefore, the PDT process can continue in the dark cycles as well as in the light cycles. The results pose the potential that this new type of fractional PDT approach significantly enhances photocytotoxic activity compared with the traditional PDT.

### 3.3. Combination Therapy Strategies

To enhance overall therapeutic efficacy, several other types of oxygen-independent therapies with potential synergistic effects have been used in combination with PDT, in particular to overcome hypoxic challenges in the tumor microenvironments. In addition, some of them can even leverage the special features in the hypoxic tumor microenvironment, such as low oxygen levels, mild acidity, and elevated H_2_O_2_ levels [95]. Here, we briefly introduce some of the most important therapies that have been designed in combination with PDT, including (1) hypoxia-targeting therapies, (2) photothermal therapies, (3) chemodynamic therapies, and (4) immunotherapies, and summarize them in Table 1.

#### 3.3.1. Hypoxia-Targeting Therapies

The emergence of hypoxia-responsive chemotherapeutic drugs provides a chance to utilize the adverse hypoxic environment to kill tumor cells via “turn corruption into wonder” methods [9]. Many of these belong to hypoxia-activated prodrugs, such as AQ4N, TPZ, TH-302, PR-104A, NLCQ-1, and SN 23,862 which can be transformed into therapeutic agents like superoxide by various intracellular reductases under hypoxia [96,97]. In contrast, in normal tissues, superoxide can be efficiently removed by oxygen. However, the use of hypoxia-activated prodrugs alone usually is not effective enough for eliminating the whole tumor, due to the hypoxic heterogeneity of tumors. Fortunately, they can compensate perfectly with PDT, according to the oxygen levels in different areas of the tumor, so that hypoxia-activated prodrugs can dominate in the area with lower oxygen levels, and PDT will be more efficient in the area with higher oxygen levels [96].

#### 3.3.2. Photothermal Therapies

Since PTT is oxygen-independent, the combination of these two phototherapeutic methods with a single nanoplatform can ensure effective co-delivery and synergistically increase the tumor killing capability. Furthermore, using nanomedicine strategies can further solve the low solubility issues of PDT agents and provide tumor-specific targeting in addition to EPR-based passive tumor targeting. Thus, many PTT/PDT dual-functional nanomedicines have been developed, such as gold nanospheres, graphene oxide NPs, WS_2_ nanosheets, and poly(dopamine) [98,99,100,115]. In addition, pretreatment with mild PTT heating to improve blood flow provides a favorable approach to relieve tumor hypoxia and regain the efficiency of PDT, and further emphasizes the clinical feasibility of the PTT/PDT combination therapy [71].

#### 3.3.3. Chemodynamic Therapies

**Figure 5:**
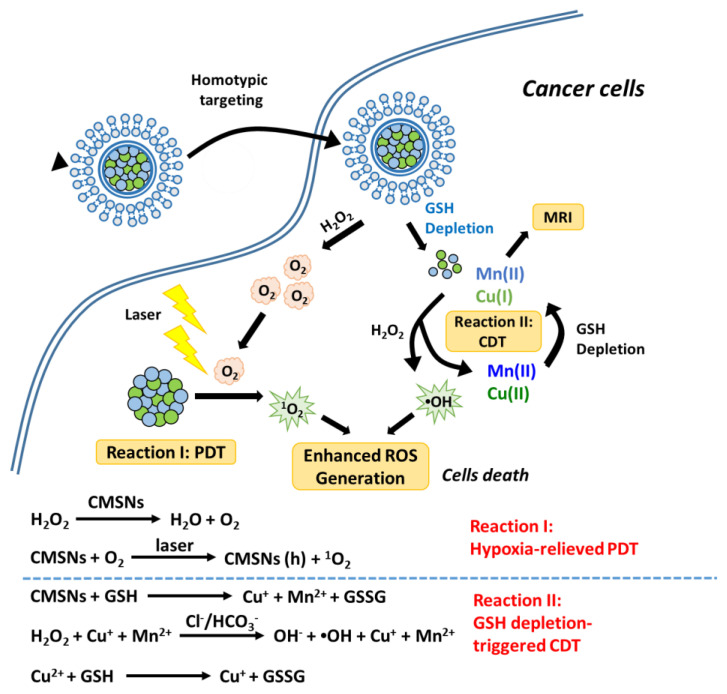
Schematic illustration of the therapeutic mechanism of copper/manganese silicate nanosphere (CMSN)-based chemodynamic therapies (CDT)/PDT combination therapy. Reproduced with permission [95]. Copyright 2019, American Chemical Society.

Generally, chemodynamic therapies (CDTs) kill tumor cells by •OH generated from in situ H_2_O_2_ by a Fenton/Fenton-like reaction, without the help of oxygen and external energy input [95]. Thus, the hypoxic tumor microenvironment with mild acidity and elevated H_2_O_2_ levels, provides a perfect working condition for CDT [104,106,107]. It has been shown that Fenton/Fenton-like reactions can simultaneously produce oxygen along with •OH, suggesting that CDT may relieve oxygen deficiency in PDT and improve its therapeutic efficiency [102,105]. In addition, the UV–Vis or NIR irradiation used in PDT can improve •OH generation efficiency in Fenton reactions to enhance the activity of CDT [103,104]. All this evidence hints that CDT will be a good partner for PDT combination therapy. For example, Liu et al. fabricated a biodegradable biomimic copper/manganese silicate nanospheres (CMSNs) for CDT/PDT synergistic therapy [95]. They showed that the CMSNs could not only exhibit effective PDT under irradiation, but also could diminish GSH levels and catalyze CDT for generating •OH at the same time (Figure 5).

#### 3.3.4. Immunotherapies

Recently, due to clinical breakthroughs, immunotherapy has become a hot area in the field of cancer treatment. Many investigations have tried to use a combination of immunotherapy and PDT to enhance the efficiency of tumor control, especially with immune checkpoint blockade strategies [108,109,110,111,112,113,114,115]. For example, Yang et al. demonstrated that combining PDT with programmed death-ligand 1 (PD-L1) antibody-based checkpoint blockade immunotherapy not only effectively inhibited tumor growth, but also decreased metastasis by potentially inducing the abscopal effect [114]. This result implies that the PDT/immunotherapy combination might have the potential to decrease the recurrence of tumors and provide better long-term control of the disease.

## 4. Emerging Trends and Outlook

In the past 20 years, significant progress has been made in nanomedicine-based PDTs, especially for enhancing the efficiency of PDT in the hypoxic tumor microenvironment. In this review, we present an overview of the state-of-the-art approaches to overcome the limits of hypoxia in PDT, mainly based on the oxygen replenishing strategies and the oxygen dependency diminishing strategies. Among the variety of approaches, some emerging trends seems to stand out.

**Figure 6:**
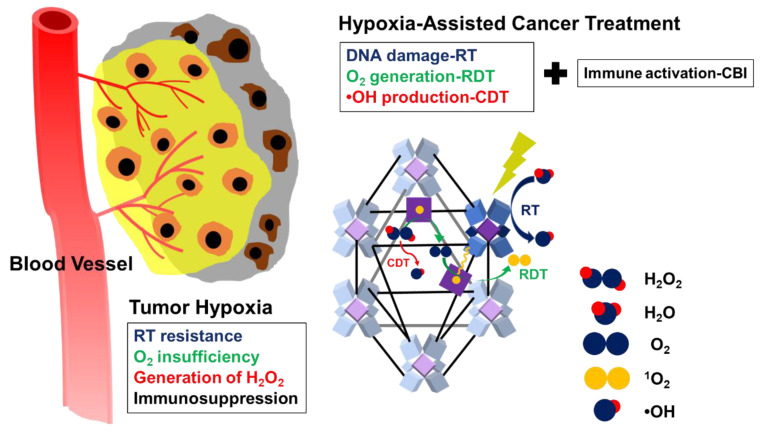
Schematic illustration of the mechanism of the nMOF as a multifunction platform for combining CDT, radiotherapy (RT), RDT, and immunotherapy for treating hypoxic tumors. Reproduced from [118]. Published by The Royal Society of Chemistry.

Firstly, the versatile nMOFs are now being widely applied within different categories of hypoxia, relieving strategies for PDT. Although MOFs have been intensely studied since the 1990s for diverse applications, the first nMOF-based PDT came as late as 2014 [116]. However, the outstanding properties caught scientists’ attention very soon. Compared to other PS nanomedicines, the porous and crystalline structures of nMOFs isolate PSs from each other to avoid self-quenching. The biodegradability of nMOFs diminish long-term toxicity when the tunable compositions and structures allow for the optimization of nMOFs for PDT applications. As mentioned previously, the great capacity of nMOFs are particularly suitable for the storage and transport of oxygen, as are PFCs for relieving tumor hypoxia [34,35,36,39]. In addition, nMOFs are also good vehicles for catalases, as is l-Arg for the generation of oxygen and NO [41,42,68]. Similarly, nMOFs also exhibit significant benefits in the application of CDT, including the availability of multiple catalytic sites and the ability to control their components. In addition, they are also effective drug delivery platforms, owing to their intrinsic porous structures for the loading of drugs, and thus can be combined with other treatment modalities easily [117]. For example, Ni et al. developed a biomimetic nMOF as a multifunction platform combining CDT, RT, radiodynamic therapy (RDT), and immunotherapy, to harnesses hypoxia for efficient tumor management (Figure 6) [118]. Thus, we can expect to see more and more nMOF-based, combination hypoxic tumor therapies. However, solubility and biocompatibility issues might limit the clinical utility of nMOFs [117].

Secondly, more and more attention is now being paid to properly incorporating immunotherapies into PDT-based tumor management. PDT has been shown to induce immunogenic cell death and stimulate innate and adaptive immune responses against tumor cells by the release of damage-associated molecular patterns and antigens from dying tumor cells [119]. The up-regulated HIF-1α has been shown to facilitate immunosuppression and immune escape by modulating lymphocytes, myeloid-derived suppressive cells, dendritic cells, and tumor-associated macrophages [9,11]. This implies that the hypoxic tumor microenvironment can hamper the induction of effective antitumor immunity via many different methods. Thus, immune checkpoint blockade strategies alone might not be sufficient to fully restore functional immune responses. In addition, the administration sequence of the combination of immunotherapy and PDT can also significantly affect the outcome. More detailed studies are still needed for designing more comprehensive and effective combination managements.

However, by surveying the recent investigation about nanomedicine-based strategies for enhancing PDT in hypoxic tumors, we find many challenges remain to be addressed still. For example, the solubility and biocompatibility issues of nMOFs and the simplified immunotherapy/PDT combination treatment protocols that might hinder their clinical usage. However, as the advance in nanomaterial modification and the knowledge about the detailed immunomodulation of PDT in hypoxic tumor microenvironments, we can expect a more valid PDT system in the near feature. Theoretically, this could be achieved by designing comprehensive therapeutic protocols via integrating the advanced nanotechnologies with other therapeutic approaches in a sophisticated time sequence. For example, oxygen and PFCs, as well as immunostimulants and cytokines, can be carried by nMOF vehicles to relieve hypoxic conditions and reverse the immunosuppressive microenvironment caused by hypoxia. These could largely increase the efficiency of the followed PDT and the stimulation of innate immune responses by the release of damage-associated molecular patterns and antigens from dying tumor cells (PDT-mediated immunogenic cell death). Then, the checkpoint blockade-based strategies can be subsequently introduced to further augment the induction of adaptive immunity.

## Figures and Tables

**Table 1:** Summary of therapies usually combined with PDT.

	Mechanism	Typical Cases	Advantages	Refs
Hypoxia-targeting Therapies	The prodrug can be transformed into therapeutic agents by various intracellular reductases under hypoxia.	˙ Hypoxia-activated prodrugs (AQ4N, TPZ, TH-302, PR-104A, NLCQ-1, and SN 23862)	˙ Leveraging the tumor microenvironment with low oxygen levels. ˙ Perfectly compensate with PDT, according to different oxygen levels in different tumor areas.	[9,96,97]
Photothermal therapies (PPTs)	Releases vibrational energy (heat) after photosensitizers are excited by light energy.	˙ Gold nanospheres, graphene oxide NPs, WS_2_ nanosheets, and poly(dopamine)	˙ It is convenient to design the irradiation method with these two phototherapeutic methods. ˙ Pretreatment with mild PTT heating to improve blood flow provides a favorable approach to relieve tumor hypoxia and regain the efficiency of PDT.	[71,98,99,100,101]
Chemodynamic therapies (CDTs)	Generating •OH from in situ H_2_O_2_ by Fenton/Fenton-like reaction.	˙ Iron-based inorganic nanomaterials; other metal-based inorganic nanomaterials (with Mn^2+^, Ti^3+^, Cu^2+^, and Co^2+^ ions); nMOFs	˙ Leveraging the mild acidic hypoxic tumor microenvironment with elevated H_2_O_2_ levels. ˙ Oxygen generated by CDT can enhance PDT efficiency, and irradiation in PDT can facilitate the •OH generation in CDT.	[95,102,103,104,105,106,107]
Immunotherapies	Augments antitumor immunity and reverses immunosuppression by immunostimulants, cytokines, and checkpoint blockades.	˙ Immunostimulants (such as Toll-like receptor ligands), cytokines (such as GMCSF), and checkpoint blockades (such as anti-PDL-1)	˙ Reverses the immunosuppressive microenvironment caused by hypoxia. ˙ Enhances the antitumor immunity induced by PDT-mediated tumor killing and tumor antigen release	[108,109,110,111,112,113,114,115]

## Data Availability

Data sharing not applicable.
